# Can Top-Down Controls Expand the Ecological Niche of Marine N_2_ Fixers?

**DOI:** 10.3389/fmicb.2021.690200

**Published:** 2021-08-18

**Authors:** Angela Landolfi, A. E. Friederike Prowe, Markus Pahlow, Christopher J. Somes, Chia-Te Chien, Markus Schartau, Wolfgang Koeve, Andreas Oschlies

**Affiliations:** ^1^Institute of Marine Sciences, National Research Council, Rome, Italy; ^2^Marine Biogeochemistry, GEOMAR Helmholtz Centre for Ocean Research, Kiel, Germany

**Keywords:** N**_2_** fixation, selective grazing, environmental controls, bottom-up control, top-down control, ecological niche, marine diazotrophs

## Abstract

The ability of marine diazotrophs to fix dinitrogen gas (N_2_) is one of the most influential yet enigmatic processes in the ocean. With their activity diazotrophs support biological production by fixing about 100–200 Tg N/year and turning otherwise unavailable dinitrogen into bioavailable nitrogen (N), an essential limiting nutrient. Despite their important role, the factors that control the distribution of diazotrophs and their ability to fix N_2_ are not fully elucidated. We discuss insights that can be gained from the emerging picture of a wide geographical distribution of marine diazotrophs and provide a critical assessment of environmental (bottom-up) versus trophic (top-down) controls. We expand a simplified theoretical framework to understand how top-down control affects competition for resources that determine ecological niches. Selective mortality, mediated by grazing or viral-lysis, on non-fixing phytoplankton is identified as a critical process that can broaden the ability of diazotrophs to compete for resources in top-down controlled systems and explain an expanded ecological niche for diazotrophs. Our simplified analysis predicts a larger importance of top-down control on competition patterns as resource levels increase. As grazing controls the faster growing phytoplankton, coexistence of the slower growing diazotrophs can be established. However, these predictions require corroboration by experimental and field data, together with the identification of specific traits of organisms and associated trade-offs related to selective top-down control. Elucidation of these factors could greatly improve our predictive capability for patterns and rates of marine N_2_ fixation. The susceptibility of this key biogeochemical process to future changes may not only be determined by changes in environmental conditions but also via changes in the ecological interactions.

## Introduction

Biological N_2_ fixation has evolved early in Earth’s history (Falkowski, 1997), when the ocean was void of oxygen (O_2_) and fixed N but rich in dissolved iron (Fe^2+^). The appearance of this process marked the beginning of the modern ocean about 2.5 billion years ago (Canfield et al., 2010). It is thus not surprising that the nitrogenase enzyme complex, which catalyzes the energy-demanding reduction of the inert N_2_ to NH_4_^+^ (Postgate, 1982), functions only under strictly anaerobic conditions and has an elevated Fe^2+^ requirement (Kustka et al., 2003). As aerobic N_2_ fixers evolved and ocean chemistry changed, diazotrophs developed numerous strategies to protect nitrogenase from O_2_, including elevated respiration rates and temporal or spatial separation of oxygenic photosynthesis from N_2_ fixation (Berman-Frank, 2001).

**FIGURE 1:**
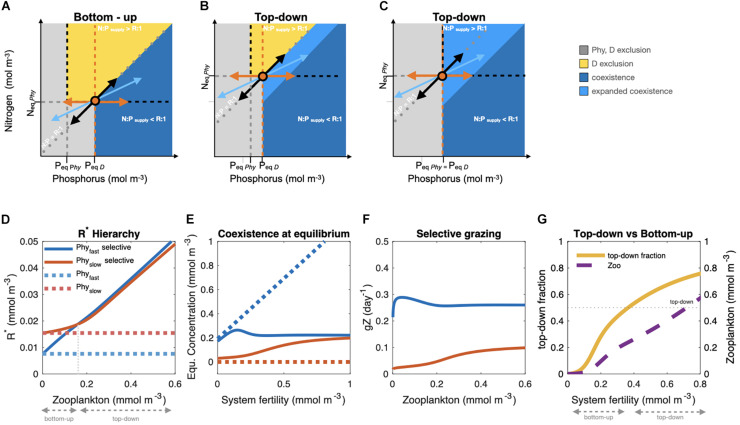
**(A)** Traditional RCT graphical approach (considering equal mortality and nutrient requirements independent from one another). In an idealized system, phytoplankton (*Phy*, black) and diazotrophs (*D*, orange) competing for nitrogen, *N*, and phosphorus, *P*, draw down nutrient concentrations to their individual equilibrium nutrient requirements (R*), here *N*_eq_ and *P*_eq_, reaching steady state (growth = mortality) on the zero net growth isoclines (ZNGI, black, and orange dashed lines). For nutrient supply below *N*_eq_, *P*_eq_ both algae cannot grow (gray region, mortality > growth). *Phy* consume nutrients (black arrow pointing toward axis origin) in their cellular proportions of R. *D* consume *P* only (orange arrow pointing left) and supply extra *N*. At steady state, total *N* and *P* consumption (blue vectors pointing toward low *N* and *P*) must be balanced by the nutrient supply (blue vectors pointing toward high *N* and P). Stable coexistence of *Phy* and *D* occurs at the interception of the ZNGIs where the growth of *Phy* is limited by *N* and that of *D* is limited by *P*. This occurs when N:P_supply_ < R, i.e., when the resource limiting the slower-growing phytoplankton is supplied in excess. Note that the region of coexistence extends all the way to the *x* axis (0 N supply) because the N added by *D* can be utilized by *Phy*. **(B)** R* depends on physiological characteristics and grazing pressure (*gZ*; Eq. 1). Accounting for selective grazing on *Phy* (*gZ_Phy_ > gZ_D_*) can increase the nutrient requirements *N*_eq_ and *P*_eq_ of the faster-growing competitor, thereby expanding the region of coexistence. **(C)** Should *P*_eq_*_*Phy*_* = *P*_eq_*_*D*_ Phy* becomes limited by *P* also for N:P_supply_ > R, allowing *D* to coexist in high *N* supply regions (see Supplementary Material for analytical derivation). **(D)** R* hierarchy: RCT predicts that in competition for the same resource, the species with the lowest R* (mmol m^– 3^) will dominate. Fast growing species (blue dashed line) dominate slowly growing ones (red dashed line) in the absence of grazing (*gZ = 0*), or selective grazing (*gZ*_fast_ = *gZ*_slow_). The inversion of the R* hierarchy occurs with selective grazing on the fastest growing organism (*gZ*_fast_ > *gZ*_slow_, solid lines). **(E)** Coexistence at equilibrium as a function of ecosystem fertility (nutrient initial conditions) for the fast (blue) and slow (red) phytoplankton in the absence of grazing or selective grazing (dashed lines) and with selective grazing (*gZ*_fast_ *> gZ*_slow_, solid lines). **(F)** Grazing rates (*g_*i*_Z*, day^– 1^) on the fast and slow phytoplankton as a function of zooplankton biomass (*Z*, mmol m^– 3^). **(G)** Top-down versus bottom-up control on R* as a function of system fertility. Top-down fraction (tdf) calculated as: $\text{tdf}=\,\sum \left(1-\frac{R_{i}{}_{\text{min}}^{*}}{R_{i}^{*}}\right)\,n^{-1}$, where for each *i*^th^ species $R_{i\,\text{min}}^{*}=R_{i}^{*}$for *g_*i*_Z* = 0, i.e., for pure bottom-up control, and *n* is the number of species. Plots **(D–G)** are based on equilibrium solutions of a 100-member ensemble of a 0D ecosystem model [nutrients (*N*), fast-growing (*Phy*_fast_) and slow-growing phytoplankton (*Phy*_slow_), and zooplankton (*Z*)] with different initial nutrient levels. *Phy*_fast_ and *Phy*_slow_ differ only in their maximum growth rate (μ_max_, 1.3 and 0.65 day^–1^, respectively) and palatability (∅_*i*_,0.7 and 0.3, respectively). All other ecosystem model parameters are equal (half saturation constant for nutrient uptake *k* = 0.5 mmol m^–3^; linear mortality *m* = 0.03 day^–1^; maximum grazing rate *g*_max_ =  0.4day^–1^; and prey half saturation constant *k*_*z*_ = 0.5 mmol m^–3^). The grazing rate per unit biomass *g*_*i*_ (m^3^ mmol^–1^day^–1^) is of the form: $g_{i}=g_{\text{max}}\,\frac{\emptyset_{i}}{\Sigma \,P\,h\,y\,i_{i}}\,\frac{P\,h\,y_{i}^{2}}{k\,z^{2}+\Sigma \,P\,h\,y_{i}^{2}}$.

The ability to fix N_2_ is associated with additional energetic costs that are generally understood to yield lower growth rates of N_2_ fixing phytoplankton as compared to their non-N_2_ fixing phytoplankton competitors. Key costs are the breaking of the triple bond and reduction of N_2_ to NH_4_^+^, and include the indirect costs required to maintain a functioning nitrogenase complex, e.g., an anaerobic intracellular environment (Großkopf and La Roche, 2012). While diazotrophs are generally considered facultative (Zehr and Capone, 2020), nitrate utilization seems to be beneficial only at rather high concentrations (above 7 μmol L^–1^, Holl and Montoya, 2005; Pahlow et al., 2013). We restrict our analysis here to conditions where diazotrophs fix N_2_. The slow-growth assumption has long guided our understanding of the competitive ability of diazotrophs (Redfield et al., 1963). It has been the basic tenet of resource competition theory (RCT) to explain coexistence patterns in idealized systems (Tilman, 1980), to understand N-inventory regulatory mechanisms on long time scales (Tyrrell, 1999; Gruber, 2004), and to simulate diazotroph activity in state-of-the-art global biogeochemical models (Ward et al., 2013; Dutkiewicz et al., 2014; Landolfi et al., 2015, 2017, 2018; Wang et al., 2019; Pahlow et al., 2020). Elevated growth rates have been reported recently for several diazotrophic species (Turk-Kubo et al., 2018), further questioning our conceptual understanding of diazotrophy. Nevertheless, the competitive advantage of N_2_ fixation in slowly growing autotrophic diazotrophs with elevated Fe-requirements should be restricted to N-limited and/or Fe-replete regions, leading to the traditional resource or “bottom-up” control paradigm of their ecological niche. This bottom-up control of autotrophic diazotrophs can be visualized using the simplified RCT graphical approach with nitrogen (N) and phosphorus (P) as limiting resources, in the absence of grazing pressure (Figure 1A).

Novel emerging data suggest that diazotrophs are distributed far more widely than the bottom-up control by N, Fe, and P predicts, spanning a wide range of ocean environments from surface to deep sea (Benavides et al., 2018) and hydrothermal vents (Mehta and Baross, 2006), from warm tropical to cold polar regions (Moisander et al., 2017; Shiozaki et al., 2017; Harding et al., 2018; Mulholland et al., 2019), from permanently oligotrophic waters of the subtropical gyres to high-NO_3_^–^ oceanic and coastal waters (Knapp, 2012; Turk-Kubo et al., 2018) to aphotic oxygen minimum zones (Löscher et al., 2014) and hypoxic basins (Hamersley et al., 2011; Farnelid et al., 2013). These oceanic environments are populated by genetically and morphologically diverse diazotrophs reflecting a large ecological diversity, from unicellular (Zehr et al., 1998) free-living (UCYN-B) and symbiont species (UCYN-A, UCYN-C) and diazotroph-diatom associations, DDA (Foster et al., 2011) to multicellular species with heterocysts (*Nodularia* and *Aphanizomenon*) and colonial forms (*Trichodesmium*), including also non-cyanobacterial diazotrophs (Riemann et al., 2010; Moisander et al., 2017). Can recent observations be reconciled with theoretical predictions?

Laboratory experiments (Knapp, 2012) as well as RCT analyses and results from numerical models of different complexity (Pahlow et al., 2013; Landolfi et al., 2015; Inomura et al., 2018) collectively suggest that autotrophic diazotrophs can still coexist with non-N_2_ fixing phytoplankton where fixed forms of N (e.g., NO_3_^–^, NH_4_^+^, and NH_3_) are available, which may be due to a combination of co-limitation by N and P (and possibly Fe or other micro-nutrients), greater N requirements for nutrient acquisition (Klausmeier et al., 2004) and the high competitive ability of diazotrophs for P (Pahlow et al., 2013). In models, with explicit representation of physiologically costly nutrient acquisition strategies, coexistence among organisms that compete for limited resources occurs for a wide range of nutrient-supply conditions (Pahlow et al., 2013, 2020; Landolfi et al., 2015; Inomura et al., 2018; Chien et al., 2020). Insight from these recent works highlights the key role of competitive interactions in setting the ecological niche of diazotrophs.

There is a growing understanding of how environmental factors affecting diazotroph physiology, with regard to temperature optima (Moisander et al., 2010), Fe requirements (Saito et al., 2011), NO${}_{3}^{-}$ tolerance (Knapp, 2012; Inomura et al., 2018), O_2_ inhibition (Stal, 2009), and competition for P (Pahlow et al., 2013; Landolfi et al., 2015), shape competition patters. There is still little knowledge, however, of how ecological interactions, such as selective mortality by zooplankton grazing and/or viral-lysis, modulate the ability of diazotrophs to compete for resources in relation to their main competitors and thereby influence their ecological niche.

## Top-Down Control Can Expand the Ecological Niche of Diazotrophs

Top-down control by grazers (Prowe et al., 2012a; Vallina et al., 2014) and mortality by viral infection (Suttle, 2007; Weitz et al., 2015) are thought to exert a major control on plankton diversity and coexistence, driving adaptation and evolution. For example, grazing can drive changes in cell size and morphology (Fenchel, 1980; Branco et al., 2020) as well as defense mechanisms (Lürling, 2021). Selective mortality, via grazing or viral-lysis, leads to increased diversity and coexistence (Thingstad, 2000), contributing, together with the spatial and temporal heterogeneity of the environment, to maintain plankton diversity (Hutchinson, 1961) on seasonal to centennial time scales (Barton et al., 2010; Tsakalakis et al., 2018; Dutkiewicz et al., 2020).

To better understand how selective mortality affects phytoplankton competition for resources and coexistence patterns, we use a simple model with one nutrient (N), a fast-growing and a slowly growing phytoplankton (Phy_fast_ and Phy_slow_) and one zooplankton (Z), and interpret our results within the RCT theoretical framework. The RCT predicts the outcome of competition and co-existence of autotrophic phytoplankton based on the R^∗^ hierarchy. R^∗^ is the resource concentration required by each phytoplankton type to reach equilibrium growth (growth = mortality). At equilibrium, each phytoplankton type draws down the ambient nutrient concentrations to its own equilibrium requirements R^∗^. It follows that different phytoplankton types can coexist only if each has at least one resource for which its R^∗^ is less than that of all others. The R^∗^ hierarchy depends on the combinations of physiological characteristics (maximal growth rate, μ_max_, and half saturation constants, *k*) and mortality terms [specific mortality *m* and grazing *gZ*, where *g* is the grazing rate per unit biomass (m^3^ mmol^–1^ day^–1^) and *Z* (mmol m^–3^) is zooplankton biomass] of each phytoplankton type, as expressed by Eq. 1 (see appendix for derivation).

(1) $${\text{R}}^{{}^{*}}=\frac{k\,\left(m+g\,Z\right)}{\mu_{\text{max}}-\left(m+g\,Z\right)}.$$

Traditionally, RCT considers the simplest case of similar mortality terms for all phytoplankton types, assuming constant specific mortality *m* and negligible effects of grazing (*gZ* = 0; Tilman, 1980). Under these simplified assumptions, the phytoplankton with the largest maximal growth rate (μ_max_; or lowest nutrient half saturation constant *k*) will have the lowest resource requirement R^∗^ at equilibrium (Figure 1D, dashed blue line), and will be the superior competitor, out-competing the slower-growing competitor (Figures 1D,E, dashed red line; Tilman, 1980). Recognizing that mortality terms may differ among phytoplankton and thereby relaxing the original RCT assumption of negligible grazing effects, we find that differential mortality, in our example mediated by selective top-down processes, but which could also include viral-mediated processes, can allow coexistence for a wider range of conditions. If zooplankton graze equally on two phytoplankton types (i.e., *gZ*_fast_ = *gZ*_slow_) the more slowly growing one will be competitively excluded as in the simplified *gZ*_fast_ = 0 case (Figure 1E, dashed lines). However, selective grazing on fast-growing phytoplankton (i.e., *gZ*_fast_ > *gZ*_slow_, Figure 1F), can change the hierarchy of R^∗^ (Figure 1D, solid lines). This allows the inferior competitor, otherwise out-competed in the absence of selective grazing, to survive and coexist (Figure 1E, compare red lines) in top-down controlled systems (Prowe et al., 2012b; analytical derivation in Supplementary Material). The relative importance of top-down control on R^∗^ is not fixed but varies, increasing with resource levels, or “fertility” of the system, as a larger zooplankton biomass can be supported (Figure 1G). With selective grazing, the effect of top-down control on R^∗^ increases with system “fertility.” It should be noted that physiologically costly defense strategies (e.g., morphological and size changes, toxicity, etc.) have associated trade-offs, implying a reduction of the maximum growth rate that would affect R^∗^ (Eq. 1) and the resulting R^∗^ hierarchy. The extended RCT framework illustrates that selective top-down control can prevent the fastest-growing organism from exploiting all of the limiting resource, expanding the niche of slower-growing species in top-down controlled regions, effectively providing for novel coexistence regimes compared to bottom-up control and non-selective mortality. Although simple, the RCT principles can help interpret phytoplankton biogeographies emerging in complex global ecological-biogeochemical models that include multiple limiting nutrients, complex grazing functions and loss terms, and circulation and mixing (Dutkiewicz et al., 2009; Ward et al., 2012, 2014).

In the specific case of autotrophic diazotrophs (*D*) competing with faster-growing non-fixing phytoplankton (*Phy*), we now extend the traditional bottom-up paradigm within the RCT graphical framework (Figure 1A) to include selective grazing on *Phy* (Figures 1B,C). This effectively expands the diazotrophs’ region of co-existence by increasing the minimum nutrient requirements, *N*_eq_ and *P*_eq_, of *Phy* (Figures 1B,C). Also, when accounting for interdependent *N* and *P* requirements (Sterner and Elser, 2002) as in chain models (e.g., Pahlow et al., 2013), selective grazing on *Phy* enlarges the niche of diazotrophs relative to bottom-up control only (Supplementary Figure 2). While competition for resources (P and Fe) has been central for explaining the diazotrophs’ spatiotemporal distribution in models (Landolfi et al., 2013, 2015, 2017; Ward et al., 2013; Dutkiewicz et al., 2014), the role of top-down control in modulating the space of diazotroph-non-diazotroph coexistence remains insufficiently explored (Wang et al., 2019). Our expanded resource-competition analysis suggests that physiological characteristics determine competitive outcomes in bottom-up controlled (nutrient scarce) environments, but as environmental resource levels increase, competition patterns become modulated by selective mortality. This can expand the ecological niche of autotrophic diazotrophs. A more comprehensive mechanistic understanding of the links between phytoplankton traits, environmental factors and ecological interactions (competition, predation, defense strategies, and mortality) is required.

### Do We Know How Diazotrophs Die?

Zooplankton grazing is considered the predominant phytoplankton mortality in the ocean (Landry and Calbet, 2004), whereas virus-mediated mortality contributes less than 10% on average (Brussaard, 2004). At low latitudes, virus-induced mortality appears to be more prevalent than at higher latitudes (Mojica et al., 2016). Under environmental stress and/or viral attack, an autocatalytic programmed cell death (PCD) has been observed in many phytoplankton species, including diazotrophs (Bidle, 2016). However, the fate of diazotroph biomass across the food web is poorly understood (Mulholland, 2007) and little is known about diazotroph mortality due to grazing, viral-lysis, or PCD and their relative importance. *Trichodesmium* is generally regarded as having low palatability for grazers (Capone, 1997), yet whether this is because of poor nutritional quality, chemical defense (toxin production), or morphological characteristics, remains unclear. Virus-mediated mortality (Hewson et al., 2004) and PCD (Berman-Frank et al., 2004, 2007) have been described as significant loss processes for this diazotroph. However, the major loss mechanisms of other diazotrophs are poorly known. Which diazotrophs (unicellular, colonial, and symbiont) are hosts for viruses, which are grazed by which size classes of grazers, micro- (<200 μm) or meso-zooplankton (0.2–20 mm), by which strategy (passive or active feeding), and at what rates is mostly unresolved. Potential traits, associated with defense strategies (morphological, physiological, and behavioral) are hardly identified and their physiological costs (trade-offs) remain mostly uncharacterized. In the following we provide a tentative synthesis of the literature reporting grazing on diazotrophs as well as a list of potential traits that could affect selective feeding (Table 1). The lack of knowledge on grazer identity, grazing rates, and traits associated with top-down processes currently limits our deterministic power in numerical models.

**TABLE 1:** Literature reporting grazing on diazotrophs.

**Diazo group**	**Predator**	**Interaction**	**TRAIT**	**Rates (day^−1^)**	**Region**	**Methos**	**References**
UCYN-A	Mesozoo (cop. *Acartia tonsa*)	Direct	Size		Baltic, WNA	PCR	Scavotto et al., 2015
	Microzoo	Indirect	Symbiosis		WTNA -Amazon	qPCR	Conroy et al., 2017
	Microzoo	Direct	Size	0.2–1	NPSG	qPCR, dilution method	Turk-Kubo et al., 2018
UCYN-B		Indirect	Aggregates		WTNA -Amazon	qPCR	Conroy et al., 2017
*Crocosphaera watsonii*		Direct	Size	0.7 ± 0.2	NPSG	Microscopy, dilution method	Wilson et al., 2017
	Microzoo	Direct	Quality		–	FlowCam	Deng et al., 2020
	Dinoflagellates	Direct	Size	0.5 ± 0.4	NPSG	IFCB – model	Dugenne et al., 2020
	Clilates	Direct	Size	0.14 ± 0.17	NPSG	IFCB – model	Dugenne et al., 2020
UCYN-C	Mesozoo	Direct			SWP	qPCR, ^15^N_2_ tracer	Bonnet et al., 2016
	Mesozoo (copepods)	Direct			SWP	qPCR, ^15^N_2_ tracer	Hunt et al., 2016
	Microzoo	No	Size	1.36–1.75	NPSG	qPCR, dilution method	Turk-Kubo et al., 2018
DDA Het1	Mesozoo	Direct			SWP	qPCR, ^15^N_2_ tracer	Bonnet et al., 2016
*Richelia-Rhizosolenia*	Mesozoo	No			SWP	qPCR, ^15^N_2_ tracer	Hunt et al., 2016
	Mesozoo (calanoid and harpacticoid cop.)	Direct			WTNA -Amazon	qPCR	Conroy et al., 2017
DDA Het2	Mesozoo (copepods)				SWP	qPCR, ^15^N_2_ tracer	Hunt et al., 2016
*Richelia-Hemiaulus*	Mesozoo	Direct			SWP	qPCR, ^15^N_2_ tracer	Bonnet et al., 2016
	Mesozoo (calanoid and harpacticoid cop.)	Direct			WTNA -Amazon	qPCR	Conroy et al., 2017
*Trichodesmium*	Mesozoo (cop. *Acartia Tonsa*)	Avoidance	Toxicity		NA	Zoo cell counts	Guo and Tester, 1994
	Mesozoo (harpacticoid)	Direct			WTNA	^14^C tracer	O’Neil and Roman, 1994
	Mesozoo	Direct			NA	Natural d^15^N zoo	McClelland et al., 2003
	Mesozoo (copepods)	Indirect	Aggregates		NP	Gut content microscopy	Wilson and Steinberg, 2010
	Mesozoo (copepods)	No			SWP	qPCR, ^15^N_2_ tracer	Hunt et al., 2016
	Mesozoo	Direct			SWP	^15^N_2_ incubations	Bonnet et al., 2016
	Mesozoo	Indirect			ETNA	Natural d^15^N zoo	Sandel et al., 2015
	Mesozoo	Direct			WTNA -Amazon	Natural d^15^N zoo	Loick-Wilde et al., 2012
	Mesozoo (harpacticoid)	Direct			Mozambique ch.	Natural d^15^N zoo	Dupuy et al., 2016
	Mesozoo (copepods)	Avoidance			Mozambique ch.	Natural d^15^N zoo	Dupuy et al., 2016
	Mesozoo (calanoid and harpacticoid cop.)	Direct			WTNA -Amazon	qPCR	Conroy et al., 2017
*Nodularia spumigena*	Mesozoo (copeod *Acartia bifilosa*)	Direct			Baltic	Natural d^15^N zoo, pigments	Meyer-Harms et al., 1999
	Mesozoo (cop. *Eurytemora affinis, A. bifilosa*)	Direct			Baltic, mesocosms	Gut content pigments	Koski et al., 2002
	Mesozoo (cop. *E. affinis., A. bifilosa*)	Direct	Toxicity		Baltic	Cell counts, toxin detection	Kozlowsky-Suzuki et al., 2003
	Mesozoo (cop. *Acartia clausii*)	Indirect			Baltic	Natural d^15^N zoo	Sommer et al., 2006
	Cladocerans	Direct			Baltic	^15^N tracer	Wannicke et al., 2013
	Micro-/mesozoo	Avoidance			Australia Estuarine	d^15^N, dilution exp.	Woodland et al., 2013
	Mesozoo (cop. *E. affinis*)	Avoidance			Baltic	^5^N_2_ tracer	Loick-Wilde et al., 2012
	Copepods, rotifers, cladocerans	Direct			Baltic	qPCR	Motwani et al., 2018
*Anabena*	Mesozoo (cop. *Acartia tonsa*)	Direct			NA, estuarine	Cell counts, dilution method	Chan et al., 2006
	Cladocerans	Direct			Baltic	^5^N_2_ tracer	Wannicke et al., 2013
*Aphanizomenon*	Cladocerans	Direct			Baltic	^5^N_2_ tracer	Wannicke et al., 2013
*Pseudoanabaena*	Cladocerans	Direct			Baltic	^5^N_2_ tracer	Wannicke et al., 2013

*Method of diazotroph detection in predator diet is reported. Grazing interactions (direct/indirect grazing or avoidance) as reported by the authors. Western North Atlantic (WNA); Western Tropical North Atlantic (WTNA); North Pacific Subtropical Gyre (NPSG); South West Pacific (SWP); North Atlantic (NA); North Pacific (NP); Eastern Tropical North Atlantic (ETNA); Imaging FlowCytoBot (IFCB).*

### Evidence of Defense Traits and Selective Top-Down Control Against Diazotrophs?

Selective top-down control can depend on prey abundance (Jürgens and DeMott, 1995; Boenigk et al., 2002), size and morphology (Armstrong, 1994), and nutritional quality (Schultz and Kiørboe, 2009), but can also be due to the host specificity of viruses. Defense strategies can be induced to reduce grazing and/or viral attack. For example, defense against grazing can include morphological and physiological traits (such as resting stages, motility), although the associated trade-offs, e.g., enhanced metabolic costs are not identified yet (Pančić and Kiørboe, 2018) and overall effects on the community level are unclear. Grazing experiments on diazotrophs are very limited (Table 1). The few observations suggest a high variability of taxon-specific grazing interactions (e.g., Wannicke et al., 2013; Deng et al., 2020) with mixed evidence of zooplankton selectivity for/against diazotrophs, based on size and morphology, extracellular environment (e.g., DOM release), nutritional quality and toxicity.

#### Size and Morphology

Size and morphology are regarded among the most important factors affecting prey selectivity (Armstrong, 1994). Zooplankton specific ingestion rates generally decrease with size of the predator (Kiørboe and Hirst, 2014), reflecting an increase in prey handling time with large sizes and complex morphologies (Wirtz, 2012). Size and morphological changes can be induced by grazing pressure and are achieved, e.g., via the formation of colonies and aggregates in phytoplankton, likely at the cost of reduced growth rates (Van Donk et al., 2011; Lürling, 2021).

In diazotrophs size, morphology, aggregation, and colony formation may thus also affect susceptibility to grazing pressure and potentially growth rates. The size spectrum of diazotrophs covers a wide range from 1 μm diameter (UCYN-A) up to *Trichodesmium* filaments of about 550 μm (LaRoche and Breitbarth, 2005; Goebel et al., 2008). Unicellular diazotrophs are part of the diet of heterotrophic and mixotrophic protists (microzooplankton; Turk-Kubo et al., 2018; Deng et al., 2020; Dugenne et al., 2020; Table 1). While large aggregates, symbionts of large organisms (>40 μm), and colonies may be protected against grazing, selective grazing on UCYN-A (Scavotto et al., 2015; Turk-Kubo et al., 2018) and UCYN-B (Scavotto et al., 2015; Conroy et al., 2017; Wilson et al., 2017; Dugenne et al., 2020) and *Trichodesmium* (Wilson and Steinberg, 2010), as part of symbionts and/or aggregates has been inferred. This suggests that aggregation and/or symbiosis may not be a deterrent for mesozooplankton grazers (Wilson and Steinberg, 2010; Hunt et al., 2016; Bonnet et al., 2018). However, given that the presence of *nifH* genes in the gut content may not unambiguously indicate direct ingestion of the diazotroph or its aggregate, conclusive evidence for direct ingestion of aggregates is scarce. In filamentous freshwater cyanobacteria, aggregation and size changes induced by the presence of grazers have been described (Cerbin et al., 2013). Triggers and costs of *Trichodesmium* colony formation and aggregation into parallel (tufts) or radial (puffs) trichomes, with presumedly different exposures to grazing, remain speculative.

The production of dissolved organic matter has been suggested to affect the size distribution, sinking speed, and accessibility to potential consumers, acting both as grazing deterrent (Liu and Buskey, 2000) or by making small cells available to larger consumers via aggregation (Passow, 2002). In *Trichodesmium*, extracellular C release has been documented and ascribed to buoyancy regulation (Villareal and Carpenter, 2003). During PCD extracellular carbohydrate release can promote the formation of sticky, fast-sinking aggregates (Bar-Zeev et al., 2013). Extracellular carbohydrates release has been observed also in *Crocosphaera* when forming aggregates (Sohm et al., 2011). The relevance of this process for reducing/increasing zooplankton grazing rates on diazotrophs is an open question that requires further elucidation.

#### Nutritional Quality

The elemental composition of phytoplankton is characterized by high plasticity compared to that of grazers (Sterner and Elser, 2002). The ingestion of nutrient deficient phytoplankton (high C:N, C:P ratios) can result in compensatory or selective feeding in zooplankton. Compensatory feeding may lead to increased ingestion of prey (to fulfill the nutrient requirement Ng et al., 2017), whereas selective feeding reduces the ingestion of prey (due to lower growth rates of grazers; Mitra and Flynn, 2006). Selection by nutritional quality may require metabolically costly and sophisticated pre-ingestion sensory abilities, as observed in marine copepods (Meunier et al., 2016). Low fatty acid content has led freshwater cyanobacteria to be considered as being of low nutritional value (Müller-Navarra et al., 2000). The range of molar C:N ratios in diazotrophs (*Trichodesmium* C:N = 4.7–8.6, LaRoche and Breitbarth, 2005; White et al., 2006; *Crocosphaera* C:N = 5.9–11.4, Tuit et al., 2004), are within the range of other phytoplankton (C:N = 5.23–9.44, Garcia et al., 2018). However, high nutritional quality in UCYN-B (*Crocosphaera*) at night (day – night C:N range = 10–6.9), driven by a light-dark N_2_-fixation pattern, has been suggested to promote night-time selective grazing by microplanktonic protists (Deng et al., 2020).

#### Toxicity

Toxin production can be induced by the presence of grazers, and lead to several adverse effects on zooplankton feeding on toxic species as a sole diet, such as reduced survival, egg production or hatching success, or induce selection against toxic species in a mixed diet (Schultz and Kiørboe, 2009; Ger et al., 2019), fostering proliferation of toxic blooms (Turner, 2014). *Anabaena (Dolichospermum*), *Nodularia, Aphanizomenon*, and *Trichodesmium* are among the toxin producing diazotrophs (Huisman et al., 2018). Early studies found that aging *Trichodesmium* (Hawser et al., 1992; Guo and Tester, 1994) and *Nodularia spumigena* (Sellner et al., 1994, 1996; Engstrom, 2000; Lundgren et al., 2012) were toxic to calanoid copepods. Hence, *Trichodesmium* has been considered as being of low palatability for most zooplankton (O’Neil and Roman, 1992), although the harpacticoid copepod *Macrosetella gracilis*, relies on *Trichodesmium* as a food source (O’Neil, 1998). Neutral or positive effects have been found also for zooplankton feeding on *Nodularia spumigena* (Hogfors et al., 2014). Some copepods show compensatory feeding (high grazing rates) on *Nodularia* (Kozlowsky-Suzuki et al., 2003). Adaptive strategies to overcome toxicity (Dupuy et al., 2016) may explain the mixed evidence of diazotroph toxicity for zooplankton.

### Model Parameterizations and Implications for Coexistence

In models a major control on coexistence of different phytoplankton functional types is exerted by the functional response that describes how predator’s ingestion depends on prey concentration (Prowe, 2012; Vallina et al., 2014). A multitude of functional responses exists that cover diverse predator-prey interactions driven by encounter, escape, selection, handling, ingestion, and digestion processes, governed by prey and predator traits and trade-offs. Three major types of functional responses (I-linear, II-hyperbolic, and III-sigmoidal) are often assumed to describe the feeding rate as function of prey concentration (Holling, 1965). However, these do not fully reproduce grazing responses observed in controlled laboratory experiments and field data (Pahlow and Prowe, 2010). The sigmoidal form (type III) stipulates proportional feeding on the most abundant prey compared to its relative contribution to total food, and may equalize *R*^∗^ values (Prowe, 2012), and is therefore often used to promote coexistence (Bates et al., 2016) and prevent extinction when diazotroph biomass is low (Landolfi et al., 2013; Ward et al., 2013). Experimental evidence for the link between feeding behavior and functional response has been found, e.g., for different copepods (Kiørboe et al., 2018). Experimental studies to derive the functional responses of zooplankton feeding strategies on diazotrophs are lacking (Table 1). Recently, a type II response has been obtained by automated imaging of grazing dynamics (Dugenne et al., 2020). In models, selective grazing can result from the combination of density dependent functional responses and phytoplankton type/size specific grazing preferences/palatability choices. Current models generally employ reduced grazing pressure for diazotrophs (selective grazing on non-fixing phytoplankton) resulting from lower palatability, size-selectivity (Dutkiewicz et al., 2020), and/or strong density dependence (e.g., type III, Landolfi et al., 2013). Diazotroph distribution and N_2_ fixation rates are very sensitive to grazing formulations. Similarly to what RCT predicts, strong selective grazing on non-fixing phytoplankton allows a greater expansion of the diazotrophs’ niche in top-down controlled nutrient rich regions, whereas weak selective grazing constrains the diazotrophs’ niche to bottom-up controlled regions in models with greater complexity in terms of multiple limiting nutrients, trophic interactions, and circulation and mixing (Supplementary Figure 3; Chien et al., 2020). Higher grazing preference for diazotrophs, relative to non-fixing phytoplankton, can result from optimized parameters in global biogeochemical models (Wang et al., 2019; Chien et al., 2020). This suggests that numerous interactions may arise in more complex models, calling for more explicit discussion and further scrutiny of the treatment of grazing formulations.

## Conclusion and Future Perspectives

The remarkable variety of growth strategies that allows diazotrophs to flourish in waters ranging from warm oligotrophic regions to cold, nutrient-rich, and highly productive systems prompts the question: What are the underlying advantages of fixing N_2_, given the associated additional energetic costs? This remarkable and widely distributed ability demands a better understanding of interdependencies between physiology and ecological dynamics (competition and predation) that set the broad ecological niches of diazotrophs. While our comprehension of physiological constraints on diazotrophs and their environmental (bottom-up) sensitivity is growing, the role of selective mortality mediated by top-down processes and/or viral-lysis in shaping their ecological niche is less clear. Relaxing the RCT’s equal mortality assumption, we identify selective mortality of faster-growing competitors as a key process for expanding the niche of autotrophic diazotrophs in nutrient rich regions. However, to date, observational evidence is limited and insufficient to support the occurrence of selective grazing against diazotrophs. Identifying traits and trade-offs associated with selective top-down control (changes in size and morphology, nutritional quality, toxicity, and DOM release) and linking them in a multi-trait perspective remains a fundamental challenge for elucidating the mechanisms that allow the ecological complexity needed for insightful model applications. Whether future environmental and ecological changes will introduce benefits or disadvantages for N_2_ fixers will depend on how these changes affect the competitive ability of diazotrophs in relation to their main competitors and predators on seasonal to centennial timescales. This suggests that we need to move beyond correlative relationships and instead establish mechanistic links between physiologically costly traits and their function in ecological dynamics. Understanding and resolving these links is key to making ecological complexity and its impact on, and interaction with, marine nitrogen fixation emerge in biogeochemical models and allowing for more reliable predictions of the future ocean.

## Data Availability Statement

The original contributions presented in the study are included in the article/Supplementary Material, further inquiries can be directed to the corresponding author/s.

## Author Contributions

This is a contribution from the “N_2_-fixation pathfinder group” at the GEOMAR Biogeochemical Modelling group. AL has written the original draft and provided the resource competition analysis. All the authors have discussed and commented-on various stages of the manuscript.

## Conflict of Interest

The authors declare that the research was conducted in the absence of any commercial or financial relationships that could be construed as a potential conflict of interest.

## Publisher’s Note

All claims expressed in this article are solely those of the authors and do not necessarily represent those of their affiliated organizations, or those of the publisher, the editors and the reviewers. Any product that may be evaluated in this article, or claim that may be made by its manufacturer, is not guaranteed or endorsed by the publisher.
