# Supplementary material for: Can Top-Down Controls Expand the Ecological Niche of Marine N2 Fixers?
Source: Front Microbiol. 2021 Aug 18;12:690200. doi: 10.3389/fmicb.2021.690200 (PMC8416505; doi:10.3389/fmicb.2021.690200)
Supplement: Supplementary file 1 [file Data_Sheet_1.pdf]

Supplementary Information

Can Top-Down Controls Expand the Ecological Niche of Marine N_2 fixers?

Landolfi A, Prowe AEF, Pahlow M, Somes CJ, Chien C-T, Schartau M, Koeve W and Oschlies A

Front. Microbiol. 12:690200. doi: 10.3389/fmicb.2021.690200

1 Competition for single or multiple nutrients

Resource competition theory [*Tilman*, 1980] has been used to describe interactions between resource availability and species composition [*Dutkiewicz et al.*, 2012]. This theory predicts that (1) At equilibrium the species with the lowest resource requirement, R^*, will outcompete the other and it will draw down the environmental resource concentration to its equilibrium requirement. (2) The resource supply ratio determines whether two species coexist or one is outcompeted at equilibrium. Here we use this theory [*Tilman*, 1980; *Dutkiewicz et al.*, 2012] expanded to include the effect of grazers [*Prowe, et al.*, 2012; *Ward, et al.*, 2014] to predict the outcomes of competition for nitrogen and phosphorus between diazotrophs and non-fixing phytoplankton in the presence of grazers. We start from equilibrium solutions of phytoplankton (P) and diazotrophs (D) growth equations neglecting advective and diffusive transport. Growth is balanced by loss terms of mortality (m) and grazing ($g_i Z$), where Z is zooplankton biomass and g_P and g_D are the zooplankton grazing rate per unit biomass for phytoplankton and diazotrophs respectively, which is a function of the maximum grazing rate (g_{max}) and the zooplankton functional response (eg: $g_i = g_{max} \frac{\phi_i}{\Sigma P_i} \frac{P_i^2}{(k_z^2 + \Sigma P_i^2)}$, ϕ_i is the preference/palatability for the i_{ith} species):

$$J_{\max}^{P} \min\left(\frac{\left[NO_3^-\right]}{k_N^P + \left[NO_3^-\right]}, \frac{\left[PO_4^{3-}\right]}{k_D^D + \left[PO_4^{3-}\right]}\right) P = m_{\mathrm{P}} P + g_{\mathrm{P}} Z P; \tag{1}$$

$$J_{\max}^{D} \left(\frac{\left[PO_4^{3-}\right]}{k_P^P + \left[PO_4^{3-}\right]}\right) D = m_{\mathrm{D}} D + g_{\mathrm{D}} Z D; \tag{2}$$

We identify the nitrate and phosphate concentration requirements at which non-fixing phytoplankton is at equilibrium:

$$[NO_3]_{eq}^{P} = \frac{k_N^P \left(m_{\mathrm{P}} + g_{\mathrm{P}} Z\right)}{J_{\max}^{P} - \left(m_{\mathrm{P}} + g_{\mathrm{P}} Z\right)} \tag{3}$$

$$[PO_4]_{eq}^{P} = \frac{k_P^P \left(m_{\mathrm{P}} + g_{\mathrm{P}} Z\right)}{J_{\max}^{P} - \left(m_{\mathrm{P}} + g_{\mathrm{P}} Z\right)} \tag{4}$$

Similarly, we derive the equilibrium phosphate requirement of diazotrophs (D):

$$[PO_4]^D_{eq} = \frac{k^D_P\,(m_{\mathrm{D}} + g_{\mathrm{D}}Z)}{J^D_{\max} - (m_{\mathrm{D}} + g_{\mathrm{D}}Z)} \qquad (5)$$

In competition for the same resource the species with the lowest nutrient requirement (R^*) will be the superior competitor and will reduce the environmental nutrient concentration down to its own (R^*) leaving insufficient nutrient for the survival of the other species [Tilman 1980]. In the absence of grazing ($gZ = 0$), phytoplankton with the largest growth rate and/or nutrient affinity (lowest R^*) will outcompete others. In the case of diazotrophs competing with non-fixing phytoplankton, their lower maximum growth rate assumption $J^D_{\max} < J^P_{\max}$ and $k^P_P \leq k^P_D$ leaves $[PO_4]^D_{eq} > [PO_4]^P_{eq}$. This implies that non-fixing phytoplankton will outcompete diazotrophs in regions where nitrate supply is high and both phytoplankton types are limited by phosphate (N:P$_{supply}$≥R; R is the elemental ratio of non-fixing phytoplankton, Fig. 1A). The two species can coexist when each phytoplankton type is limited by a different nutrient. Coexistence is thus possible when non-fixing phytoplankton is limited by nitrate and diazotrophs are limited by phosphate. This occurs at the intercept of the zero net growth isoclines (ZNGI, Tilman 1980) (Fig. 1A) [Landolfi et al., 2015]. For N:P$_{supply}$< R nitrate concentration will be drawn down by non-fixing phytoplankton to their equilibrium requirement $[NO_3]^P_{eq}$ and environmental phosphate concentration will be drawn down to the equilibrium requirement $[PO_4]^D_{eq}$ of diazotrophs allowing for coexistence.

However, when grazing is taken into account then R^* increases with grazing pressure (suppl. Equ. 3, 4, 5). This leaves the possibility that grazing changes the hierarchy of R^* allowing the inferior competitor to survive and coexist with the superior competitor for nutrients (Fig. 1D, E). In the specific case of diazotrophs competing with non-fixing phytoplankton, even where nitrate supply is high and both phytoplankton types are limited by phosphate, there can be a possibility that $[PO_4]^D_{eq} \leq [PO_4]^P_{eq}$ (Fig. 1B,C) expanding coexistence regions, making diazotrophs survive in N:P$_{supply}$ ≥R regions. From Equation 4 and 5 we find that this can occur if $g_{\mathrm{P}} > g_{\mathrm{D}}$, i.e. if selective feeding on non-fixing phytoplankton occurs. It should be noted that active defence strategies for grazing reduction (such as colony formation, morphological changes, toxicity, etc.) are expected to be physiologically costly and have an associated trade-offs in terms of reduction of maximum growth rate or nutrient requirement. This implies that active defence mechanisms also affect R* hierarchy and regions of co-existence.

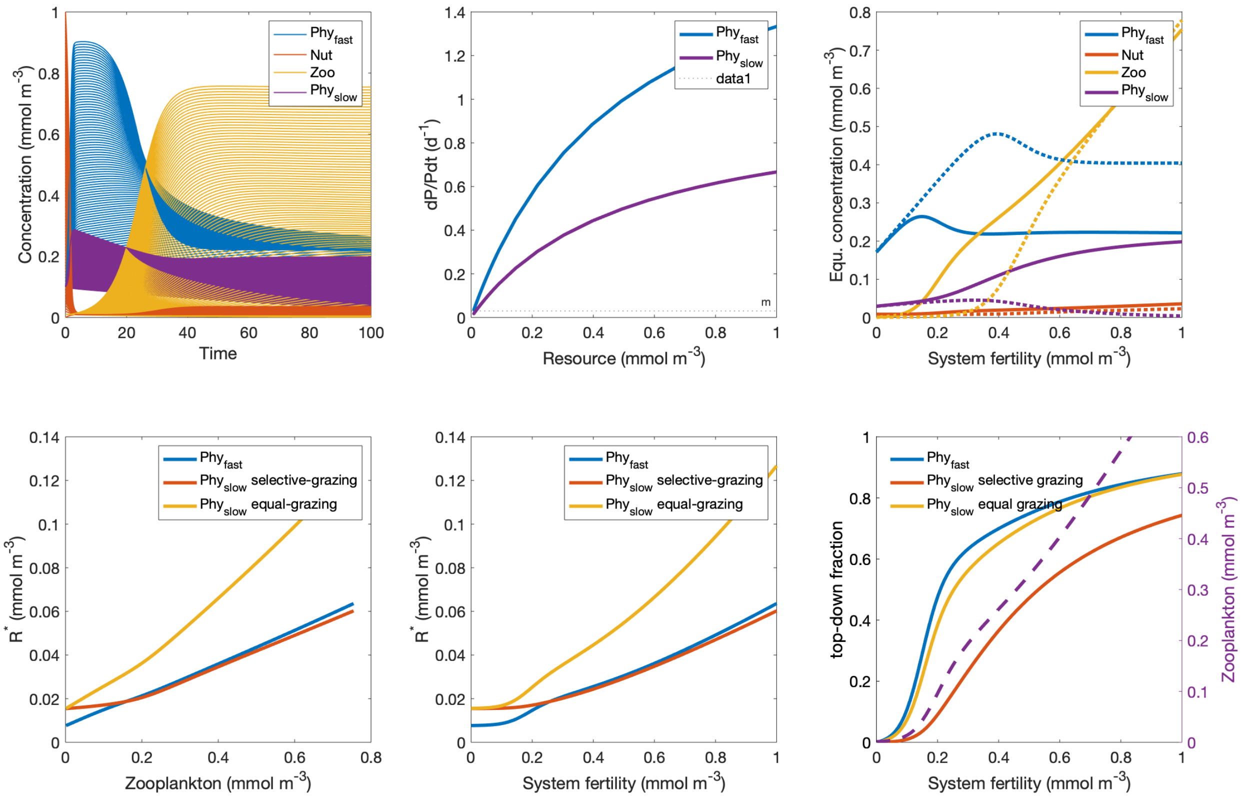

Fig. S 1: 0D ecosystem model 100 ensemble member (Nutrients (N), Phy_{fast}, Phy_{slow}, Zooplankton (Z)) with differing initial nutrient levels and grazing rates, g_P and g_D. (a) Phy_{fast}, Phy_{slow}, Zooplankton and Nutrients ($mmol\ m^{-3}$) concentration for each ensemble member. (b) Phy_{fast}, Phy_{slow} growth (d^{-1}) rate as function of resource level ($mmol\ m^{-3}$). (c) Equilibrium concentrations ($mmol\ m^{-3}$) as function of the resource level (initial conditions) ($mmol\ m^{-3}$); dashed lines are equilibrium solutions for model runs with same grazing rates for both phytoplankton types i.e.: $g_P = g_D$. (d) R^* ($mmol\ m^{-3}$) as a function of zooplankton biomass (Z, $mmol\ m^{-3}$), for 3 phytoplankton configurations: fast-growing (blue), slow-growing with same grazing rates $g_P = g_D$ and slow-growing with selective grazing $g_P > g_D$. (e) R^* ($mmol\ m^{-3}$) as a function of initial resource levels (N, $mmol\ m^{-3}$), for 3 phytoplankton configurations: fast-growing (blue), slow-growing with same grazing rates $g_P = g_D$ and slow-growing with selective grazing grazing $g_P > g_D$. (f) Top down fraction as a function of ecosystem fertility (initial resource levels). Phy_{fast}, Phy_{slow} differ only in their maximum growth rate ($\mu_{max} = 1.3$ and 0.65, d^{-1}, respectively) and grazing g_P and g_D. All other ecosystem model parameters are equal (half saturation constant for nutrient uptake $k = 0.5\ mmol\ m^{-3}$; linear mortality $m = 0.03\ d^{-1}$; maximum grazing rate $g_{max} = 0.4\ d^{-1}$; prey half saturation constant $k_z = 0.5\ mmol\ m^{-3}$. The grazing rate per unit biomass g_i ($m^3 mmol^{-1} d^{-1}$) is of the form: $g_i = g_{max} \frac{\phi_i}{\Sigma P_i} \frac{P_i^2}{(k_z^2 + \Sigma P_i^2)}$, ϕ_i is the palatability (0.7 and 0.3 for fast-growing (blue) and slow-growing with selective grazing grazing (red), respectively).

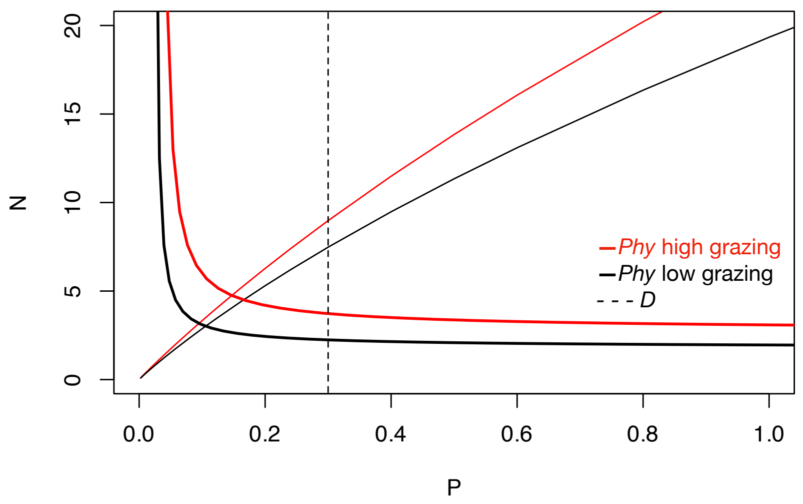

Fig. S 2: Stable coexistence of Phy (thick black line) and D (black dashed) occurs at the interception of the ZNGIs where the growth of Phy is limited by N and that of D is limited by P, also considering the dependency of N_{eq} on P availability [Sterner and Elser, 2002] as in chain models [e.g., Pahlow et al., 2013, 2020], where the ZNGI are curved owing to the N_{eq} requirement depending on P availability. Thin lines are the upper boundaries of the N supply concentrations for D and Phy coexistence, for lower (black line) and 20% higher (red line) mortality by grazing on Phy. D will be excluded for P below their P_{eq} (left of their ZNGI, black dashed line) and above the thin lines. Strong top-down control (high grazing on Phy, red lines) affects N_{eq} and P_{eq} of Phy and expands the region of coexistence area between the red and black thin lines.

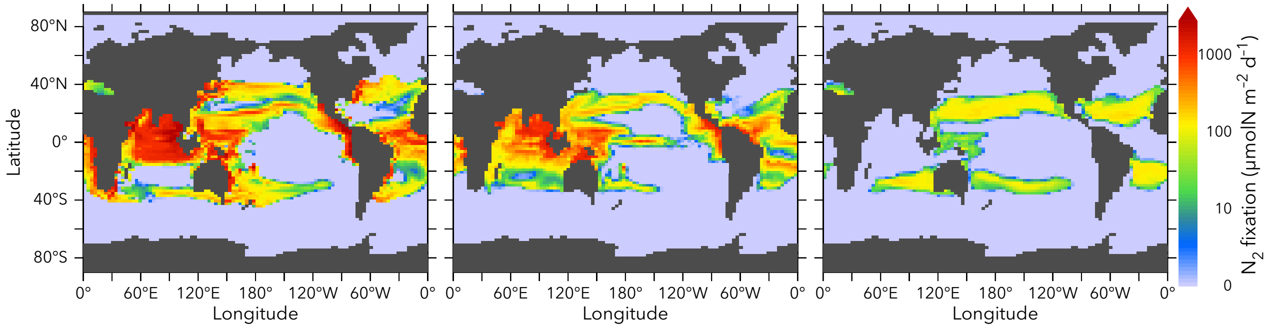

Fig. S 3: Effect of grazing preferences in a state-of-the art global biogeochemical model with a type II functional response [Chien et al., 2020] in the distribution of N_2 fixation rate in a sensitivity model experiments with (a) selective grazing on phytoplankton, (b) no selective grazing (c) selective grazing on diazotrophs.

References

Chien, C.-T., Pahlow, M., Schartau, M., and Oschlies, A. (2020). Optimality-based non-Redfield plankton–ecosystem model (OPEM v1.1) in UVic-ESCM 2.9 – Part 2: Sensitivity analysis and model calibration. Geosci. Model Dev. 13, 4691–4712. doi:10.5194/gmd-13-4691-2020.

Dutkiewicz, S., B. A. Ward, F. Monteiro, and M. J. Follows (2012), Interconnection between nitrogen fixers and iron in the Pacfic Ocean: Theory and numerical model. *Glob. Biogeochem. Cycles 26*, doi:10.1029/2011GB004039.

Landolfi, A., H. Dietze, W. Koeve, A. Oschlies (2015), Overlooked runaway feedback in the marine nitrogen cycle: the vicious cycle. Landolfi, A., W. Koeve, H. Dietze, P. Kähler, and Oschlies A. (2015). A new perspective on environmental controls of marine nitrogen fixation. *Geoph. Res. Lett. 42(11)*, 4482-4489.

Pahlow, M., C.-T. Chien, L. A. Arteaga and A. Oschlies (2020). Optimality-based non-Redfield plankton-ecosystem model (OPEMv1.1) in the UVic-ESCM 2.9. Part I: Implementation and model behaviour. Geosci. Model Dev. Discuss. 2020. doi: 10.5194/gmd-2019-323. In press.

Pahlow, M., H. Dietze and A. Oschlies (2013). Optimality-based model of phytoplankton growth and diazotrophy. Mar. Ecol. Prog. Ser. 489: 1–16. doi: 10.3354/meps10449.

Prowe, A. E. F., Pahlow, M., and Oschlies, A. (2012). Controls on the diversity–productivity relationship in a marine ecosystem model. Ecol. Model. 225, 167–176. doi:10.1016/j.ecolmodel.2011.11.018.

Sterner, R. W., and Elser, J. J. (2002). Ecological stoichiometry: the biology of elements from molecules to the biosphere. Princeton Univ. Press.

Tilman, D. (1980) Resources: A graphical-mechanistic approach to competition and predation. *The American Naturalist 116*, 361-393, doi:10.1086/ 283633.

Ward, B. A., Dutkiewicz, S., and Follows, M. J. (2014). Modelling spatial and temporal patterns in size-structured marine plankton communities: top–down and bottom–up controls. J. Plankton Res. 36, 31–47. doi:10.1093/plankt/fbt097.
